# Simulation and Experimental Study of a Piezoelectric Stack Energy Harvester for Railway Track Vibrations

**DOI:** 10.3390/mi14040892

**Published:** 2023-04-21

**Authors:** Zhaowei Min, Chengwei Hou, Guangdong Sui, Xiaobiao Shan, Tao Xie

**Affiliations:** State Key Laboratory of Robotics and System, Harbin Institute of Technology, Harbin 150001, China

**Keywords:** piezoelectric stack, energy harvester, railway system, rail track vibration, low frequency

## Abstract

As one of the most important modes of transportation, the safety of running trains and railway tracks is significant. It is essential to power sensors that detect and track health in remote areas. The vibration energy of the track structure is enormous, stable, and not limited by weather factors such as the sun and wind. A new type of arch beam piezoelectric stack energy harvester for railway systems is studied in this paper. Through simulation analyses and experimental verification of the energy harvester, the influences of external resistance, load, pre-stress, and load frequency on the energy harvesting performance of the piezoelectric energy harvester are discussed. When the frequency is less than 6 Hz, the energy capture efficiency is greatly affected by the frequency. When the frequency exceeds 6 Hz, the frequency has little effect and the load dramatically affects the energy capture efficiency. The pre-stress has little effect on the energy capture efficiency, but there is an optimal value at 4.5 kN. The energy harvester has an output power of 193 mW, a weight of 912 g, and the energy density can reach 211.8 μW/g. These results can provide a reference for subsequent experiments in the actual environment.

## 1. Introduction

**Figure 1:**
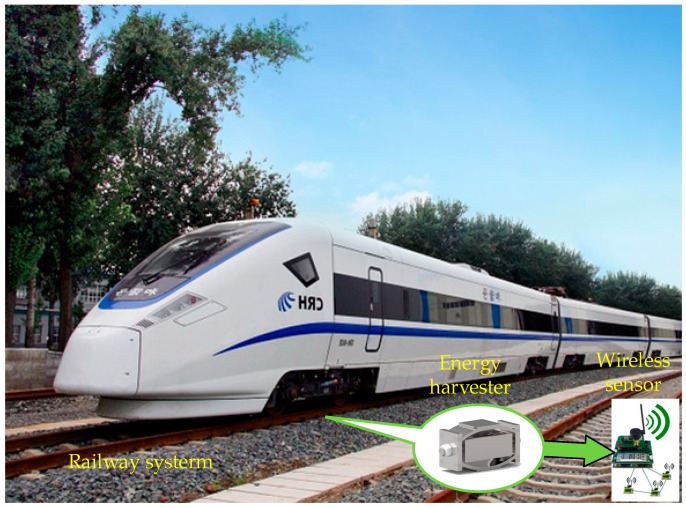
Schematic diagram of vibration energy harvesting system applied to railway track.

As one of the most important modes of transportation, railways are widely used worldwide, and developments in high-speed and intelligent railways are growing rapidly [1]. By the end of 2021, the total length of railways in China exceeded 150,000 km, of which more than 40,000 km was high-speed railway with speeds exceeding 200 km/h [2]. Therefore, it is essential to ensure the safety of trains. Many wireless sensors are installed along the railway and connected to a network to detect the health status of the critical infrastructure of tracks and bridges [3,4,5]. However, the power supply for wireless sensors is a problem that still needs to be solved, especially in remote areas [6,7]. Previous studies have proven that capturing the mechanical vibrational energy of track structures is feasible by using various energy harvesters as power sources for wireless sensor networks [8,9]. In addition, the track structure’s vibrational energy is significant, stable, and not limited by weather factors such as the sun and wind [10,11]. Figure 1 shows a schematic diagram of a track vibration energy harvester supplying energy to the wireless sensor networks. Research and industry are paying more attention to vibrational energy acquisition at railway tracks. The primary forms of vibrational energy harvesting are triboelectric energy harvesting, electrostatic energy harvesting [12,13,14], hydraulic energy harvesting, piezoelectric energy harvesting [15], and electromagnetic energy harvesting [16,17,18].

A piezoelectric energy harvester utilizes the direct piezoelectric effect of piezoelectric materials, i.e., the charge change caused by compression deformation of piezoelectric materials [19,20,21]. Previous studies have focused on patch-type piezoelectric energy harvesters [22] and cantilever-type piezoelectric energy harvesters [23,24,25], which are usually connected to railways or railway bridges. A patch-type piezoelectric energy harvester mainly collects energy from the track or railway bridge deformations caused by passing trains. A cantilever piezoelectric energy harvester captures the train vibrational energy by matching the resonant frequency of the energy harvester with the central frequency of the rail or railway bridge vibrations. Yuan et al. [26] designed a drum sensor set installed under a sleeper, effectively utilizing the significant amount of track vibration force. Wang et al. [27] developed a piezoelectric circular film array, which improved the output power and broadened the operating frequency bandwidth, and is expected to be applied in the high-speed railway system. The design combined stacked and cymbal transducers. Hou et al. [28] proposed a piezoelectric vibration energy harvester where the harvester and the floating concrete slab of steel springs were in series arrangement. The system had a small volume, was lightweight, had a strong carrying capacity, had a higher energy recovery efficiency, and could make full use of the train-induced steel and spring protection forces. These achievements have extensively promoted the development of energy harvesting technology in the railway system. In addition, compared with electromagnetic energy harvesters, piezoelectric energy harvesters have the advantages of a simple structure, a small size, no electromagnetic interference [29], and are easier to miniaturize and integrate with wireless sensor nodes. Fu et al. developed a double-sided fixed piezoelectric vibration energy harvester installed on underground trains in 2019 [30], which could output multiple voltages over a wide frequency range with a maximum voltage of up to 60 V. Song et al. studied a piezoelectric energy catcher based on a cantilever structure installed on a superconducting maglev train [31]. The experimental results showed that the output voltage of the harvester increased with an increase in the vibration frequency, and the maximum voltage exceeded 6 V. Cho et al. [32] also proposed a piezoelectric energy harvester based on a cantilever mechanism to power the safety monitoring sensor of the train.

In recent years, piezoelectric stack energy harvesting devices have attracted increasingly more attention [33,34]. Yalei Cao et al. [35] proposed a piezoelectric tube stack energy harvester which could be placed at the bottom of the rail to collect vibrational energy from the vertical displacement caused by train motion. The energy harvesting performance of the piezoelectric tube stack energy harvester was experimentally investigated and the results were consistent with the theoretical results of a simplified model of the piezoelectric tube stack energy harvester. The energy predicted by the theoretical model when the train passes was 82.16 mJ, and the power measured by the prototype was 68.38 mJ at the optimal resistance. The energy harvesting performance could be improved by using piezoelectric tube stacks with small areas, multiple piezoelectric layers, and thick piezoelectric layers. The energy harvester proposed by Guansong Shan had a maximum output power of 495.74 mW and an average power of 43.58 mW under the conditions of an input RMS acceleration of 0.7 g, 18 Hz, and an optimal load impedance of 150 Ω. Compared with the most advanced piezoelectric energy harvester applied in railways, it performed well [36]. As for the energy harvester proposed by Jianjun Wang et al. [9], under the actual steel spring fulcrum force level, the maximum average power of the prototype could reach 500 mW. In addition, the full average power could achieve values on the order of 1 W by designing an appropriate piezo-stack radius and appropriate piezo-stack number. Weiqiang Sheng et al. [37] proposed an efficient dynamic amplifying piezoelectric energy harvester whose energy output was up to 0.55 W under the excitation of low-frequency harmonic acceleration per unit amplitude. A high energy output was achieved by combining dynamic amplifiers with several composite piezoelectric transducer units with d33 modes. The prominent feature of the piezoelectric energy harvester designed by Wenqi Hou et al. [38] was the force amplification mechanism composed of a cover plate and bending plate, which was helpful for improving the energy harvesting efficiency. One hundred and forty-four stacks were arranged on thirty-six units, and the total energy power obtained could reach 31.4 kJ. Recent research on railway vibration energy harvesting shows that piezoelectric vibrations are being used more often. They have the advantages of a high efficiency, immense power, and low environmental requirements. However, the piezoelectric material is brittle, so the piezoelectric stack should be fully protected to cope with the impact of large loads. Based on the high energy capture efficiency of a piezoelectric stack, an arch beam has been designed to convert the downward pressure generated by rail vibrations into the tension of the pull rod so that the pressure plate can cause strain on the piezoelectric stack. Therefore, the piezoelectric stack is fully protected to ensure the high-power energy in complex working conditions is utilized and to maintain the service life of the piezoelectric stack.

The new contributions of this paper include: (1) An arched beam which converts the compressive stress from the track to tensile stress on the tie rod, further buffering the pressure. A design scheme for the piezoelectric stack is proposed. The piezoelectric stack’s transverse arrangement reduces the device’s vertical height. The feasibility of the design is verified by the prototype of a piezoelectric stack energy harvester. (2) A simulation experiment is carried out on the energy capture device under railway displacement to further verify the performance of the energy capture device. (3) The influence of the critical parameters of the piezoelectric stack on the energy harvesting performance of the energy harvester is studied, and suggestions for further optimization are put forward.

Moreover, Section 2 establishes a quasi-static rail model based on the Winkler beam and the energy harvester design. Section 3 introduces an analysis of the energy harvester simulation and experimental verification. We discuss the influence of external resistance, load size, pre-stress size, and load frequency on the energy harvesting performance of the piezoelectric energy harvester. Section 4 summarizes the main results of this work.

## 2. Modeling Rail and Designing Energy Harvester

**Figure 2:**
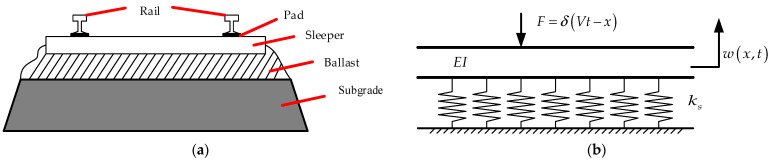
Example of a railway track structure: (**a**) railway track profile; (**b**) a simplified model of the railway structure.

The primary source of low-frequency vibrations in railway tracks is the quasi-static load passing through the train [39,40]. This incentive mechanism is characterized by a significant vibration level at the so-called train load frequency [41] through the train’s axis sequence [42]. Figure 2a shows a schematic cross-section of a typical ballasted railway. The system consists of two steel rails connected to concrete sleepers by clamps and rail pads; the sleepers on the ballast and roadbed form a unit track length distribution rail bed stiffness, *k_s_*. The low-frequency vertical vibration of the railway (<30 Hz) is the focus of this paper. In this frequency range, the track vibration is mainly caused by the quasi-static moving axle load. This is independent of vehicle and track dynamics, whereas the track vibration due to dynamic excitation causes track and wheel irregularity only at higher frequencies. Therefore, the model ignores the motional effects and uses a quasi-static railway model. Each train wheel can be assumed to act as a steady point force moving on the beam at the train speed *V*. The higher-order effects such as shear deformation are only significant above about 500 Hz; thus, the Euler–Bernoulli beam theory can be used to study the rails [43]. The distance between the two sleepers (0.65 m) is much smaller than the bending wavelength of the track at these low frequencies, and on this basis, the rail can be considered to be an equivalent Winkler foundation. 

**Figure 3:**
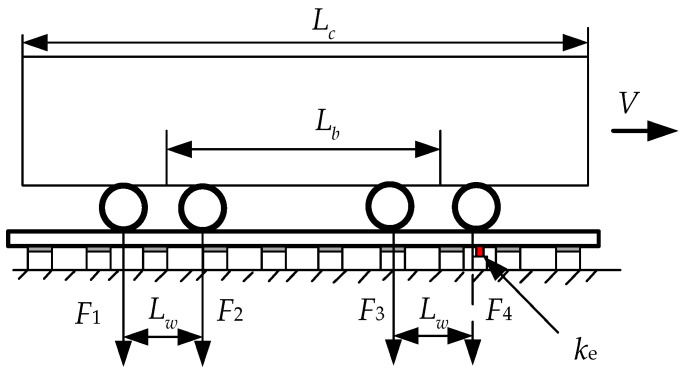
Example of the load applied by each wheel of a carriage.

An example of a single wheel load is shown on the model of a monorail system in Figure 2b. *E* is Young’s modulus of the rail, *I* is the moment of inertia of the rail, *EI* is the flexural stiffness of the fence, *ρ* is the mass per unit length of the rail structure, *w*(*x,t*) is the vertical deflection of the rail, *δ* is the Dirac function, and *x* is the horizontal position of the wheel concerning any reference point. Figure 3 shows a typical single car of a train, where *L_c_* is the carriage length, *L_w_* is the distance between the two wheels on the bogie, and *L_b_* is the distance between the center points of the two bogies. The equivalent stiffness of the piezoelectric energy harvester is *k_e_*, which can be obtained by experimental verification of the force displacement curve. The equation of motion of a single car acting on the track is given in (1):

(1) $$EI\frac{\partial^{4}w\left(x,t\right)}{\partial x^{4}}+\left(k_{s}+k_{e}\right)w\left(x,t\right)=-\sum_{i=1}^{4}F_{i}\delta \left(Vt-x-d_{i}\right)$$

where *t* is the time, *F_i_* is the force on the wheel, *d*_1_ = 0, *d*_2_ = *L_w_*, *d*_3_ = *L_b_*, and *d*_4_ = *L_w_* + *L_b_*. 

**Figure 4:**
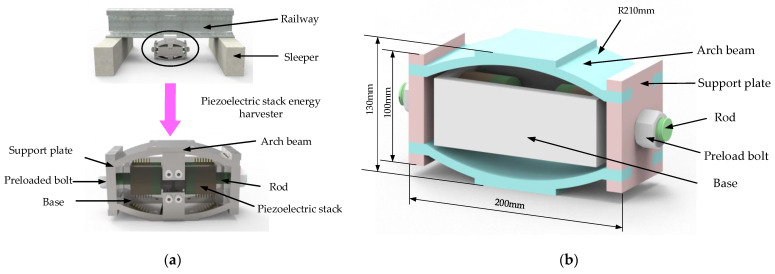
Schematic diagram of a piezoelectric energy harvester. (**a**) Schematic diagram of the energy harvester placed under the rail; (**b**) schematic diagram of a three-dimensional model of the energy harvester.

The designed arch beam piezoelectric stack energy harvester is shown in Figure 4. The energy harvester system comprises the arch beam, support plate, preload bolt, rod, base, pressure disk, and piezoelectric stack, which are mainly used to capture the energy of the vehicle-induced vibrations and effectively capture the power generated by the railway vibrations. The energy harvester is placed below the rail. When the arch beam receives vertical vibrations and impact from the rail, it deforms and generates lateral outward thrust on the sliding support plate. The support plate, the preload bolt, the tie rod, and the pressure disk become one unit, so that the piezoelectric stack is pressurized and the energy generated by the vibrations is captured. The arch beam can avoid the direct impact of the piezoelectric stack when it is affected by the pulse of the rail, so the piezoelectric stack is well protected. At the same time, the force can be transferred to the piezoelectric stack through the support plate, rod, and pressure disk, so that the energy harvester can efficiently capture energy while ensuring the safety of the piezoelectric stack.

**Figure 5:**
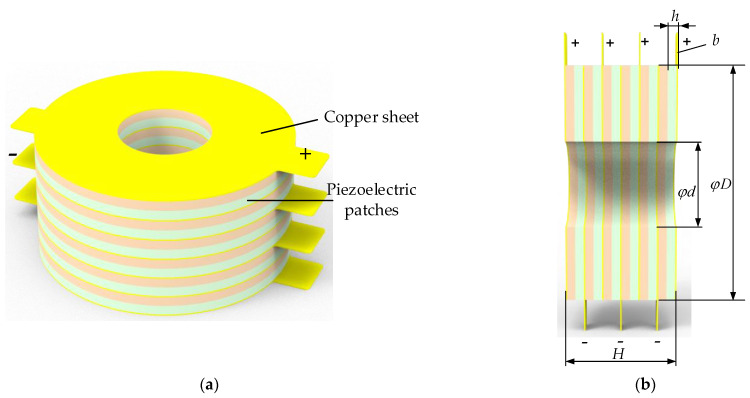
Schematic diagram of the piezoelectric stack. (**a**) Schematic diagram of piezoelectric stack structure; (**b**) schematic diagram of the piezoelectric stack size.

The piezoelectric stack in the energy harvester is arranged in two horizontal symmetrical ways, which can balance the stress and deformation of the arched beam. Each group of the piezoelectric stack is composed of 12 annular piezoelectric plates in parallel. Each piezoelectric stack consists of two piezoelectric plates in series as a unit and then six units in parallel. The distribution resistance can be reduced when the piezoelectric plate is completely parallel, but the output voltage is not high. When the load is large, the complete series mode will generate thousands of volts of voltage, leakage and other situations will occur, and there are also problems in the subsequent processing. Series and parallel hybrid use not only ensures that the voltage can reach more than 200 V, but also ensures a low optimal resistance value. The total height of the piezoelectric stack is *H* = 27.9 mm, the thickness of the piezoelectric plate is *h* = 2 mm, the thickness of the copper plate is *b* = 0.3 mm, the inner diameter is φd = 20 mm, the outer diameter is φD = 55 mm, the weight is 456 g, and the volume is 57,818 mm^3^. Its structure is shown in Figure 5. 

## 3. Simulation and Experimental Discussion of Energy Harvester

**Figure 6:**
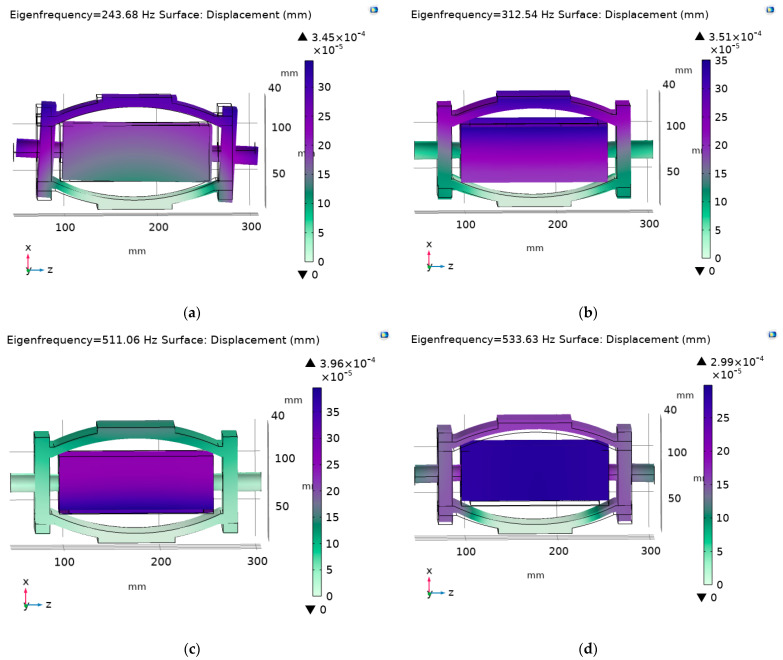
Modal cloud image of a piezoelectric energy harvester. (**a**) The first mode; (**b**) the second mode; (**c**) the third mode; and (**d**) the fourth mode.

Finite element simulation is a standard numerical method in scientific research and engineering. It has the advantages of a wide application range, flexible application, and strong practicability, and it can effectively improve the efficiency of scientific research. COMSOL Multiphysics is a mature and widely used finite element analysis software for fully coupling multi-physical fields. Coupling between solid mechanics and an electric field is widely used. COMSOL Multiphysics was used to simulate and analyze the 3D model. The piezoelectric stack uses a piezoelectric ceramic (PZT) brittle material; thus, brittle failure easily occurs under an impact load. Therefore, the deformation of the arch beam after the impact load plays a role in buffering the force on the piezoelectric stacks and filters the high-frequency vibrations, thus protecting the piezoelectric stacks of brittle material. In the modal analysis stage, a three-dimensional model will be introduced into COMSOL. Lead zirconate titanate (PZT-8) is used in the energy harvester and structural steel is used for the arch beams and other structural materials. Multiphysics selects the piezoelectric effect. The mesh is divided into a maximum of 14.3 mm and a minimum of 1.03 mm, with a free tetrahedral shape. The eigenfrequency is selected for study and the solution is set to solver configurations: eigenfrequency. The other options are set to default. The natural frequencies of the energy harvester are as follows: first order: 243.68 Hz, second order: 312.54 Hz, third order: 511.06 Hz, and fourth order: 533.63 Hz. This is much larger than the frequency of the track impact vibrations, so in the process of use, the overall resonance will not be generated. Only the quasi-static impact vibrations of the rail caused by the vehicle are collected so that the service life of the energy harvester is long. A modal cloud diagram is shown in Figure 6.

**Figure 7:**
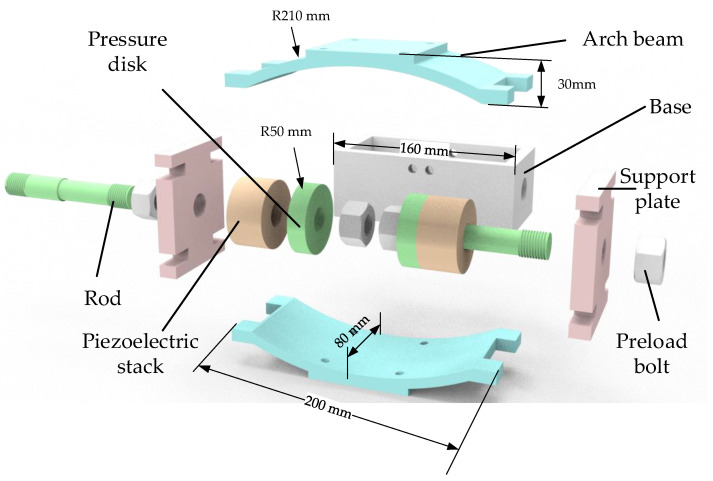
Configurations of the mode piezoelectric energy harvester.

**Figure 8:**
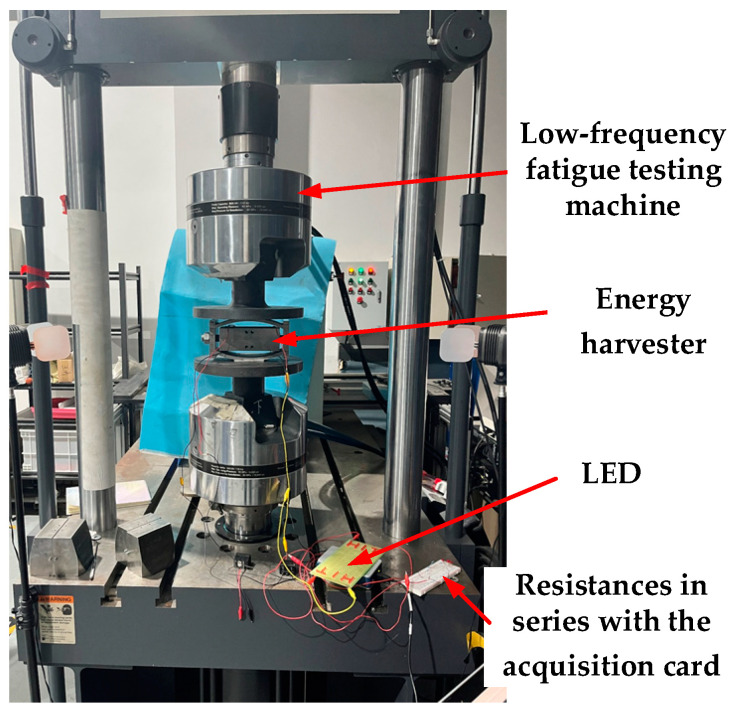
Schematic diagram of the installation position of the experimental device for testing the performance of the energy harvester.

The energy harvester can meet the use requirements in the railway vibration environment through the above simulation analysis. The experimental prototype is made and assembled through mechanical processing. The parts of the energy harvester are shown in Figure 7. At the same time, a pressure test was carried out on a MTS 370-10 universal fatigue test machine [44]. The MTS 370 load frame uses a lightweight design with a high stiffness cross head to improve the natural frequency of the system and a high precision machining frame column to ensure the long-term neutrality of the system. The actuating cylinder rating is 50 kN, the actuating cylinder dynamic stroke is 100 mm, and the frame weight is 635 kg under the rated load. At 1–20 Hz, the sine motion load is less affected by the frequency. The overall height is 2588 mm and the frame column spacing is 533 mm. This meets the specifications required by the experiment. The energy harvester was placed on the MTS test machine, the parameters of the test machine were adjusted, the National Instruments (NI) data acquisition card was connected, and the data of the acquisition card were stored on a personal computer. The installation of the energy harvester and data acquisition card is shown in Figure 8. 

**Figure 9:**
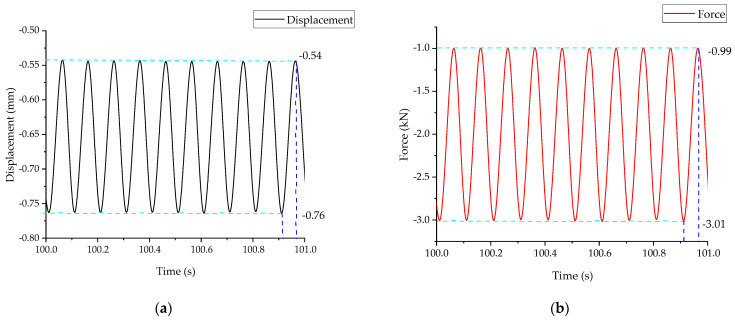
Schematic diagram of force and displacement time curves: (**a**) displacement–time curve; (**b**) force–time curve.

The time domain diagram of the collected MTS output pressure and displacement is shown in Figure 9. The displacement and pressure curves are well integrated; when the maximum pressure is 3.01 kN, the maximum displacement is 0.76 mm. When the minimum pressure is 0.99 kN, the minimum displacement is 0.54 mm. Therefore, the equivalent stiffness of the energy harvester is 10 MN/m. The stiffness of the trackpad varies significantly between the track with ballasts and the ballast-less track. The stiffness of the ballast-less trackpad is in the range of 20–30 MN/m, while that of the ballast trackpad is in the range of 90–120 MN/m. Therefore, the coupling of the equivalent stiffness of the energy harvester should be considered. 

**Figure 10:**
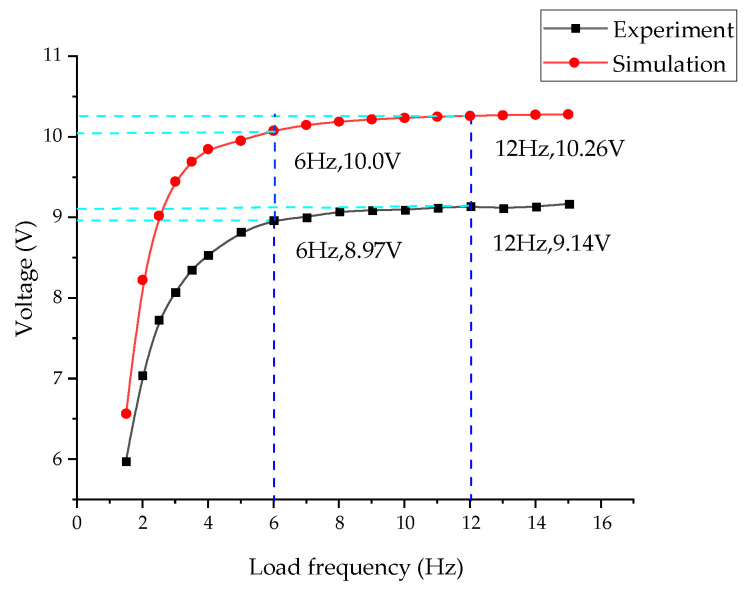
Influence of load frequency on the output voltage.

When the simulation frequency affects the voltage, the pre-setting is consistent with the modal analysis. In solid mechanics, the boundary load pre-stress, *F*_1_, is 2000 N, and the perturbation load, *F*_0_, is 2000 N. The frequency settings are as follows: Study: Frequency Domain, Pre-stress; step 1 is the default; and step 2: Frequency Domain Perturbation Frequencies: range (1, 0.5, 4) range (5, 1, 15). The influence of load frequency on the energy harvesting effect of the energy harvester under a pre-stress of 2 kN and a sinusoidal disturbance load of 2 kN was analyzed, and the open-circuit RMS (root mean square) voltage is shown in Figure 10. Due to the machining and assembly errors of the energy harvester and the errors of the MTS testing machine, the voltage generated by the experiment is smaller than that of the simulation results. At 1 Hz, a small voltage is produced; at 1–6 Hz, with the increase in frequency, the piezoelectric voltage increases more quickly; and in the 6–12 Hz range, the voltage also increases with the increase in frequency. However, the influence on the voltage is reduced at 12–15 Hz, and the frequency has a minor effect on the voltage at 15 Hz. Thus, the voltage peaks at 9 V (10.3 V in the simulation). The frequency range of railway impact load is 3–8 Hz [45], and the energy capture frequency of the energy harvester meets the scope of use. 

**Figure 11:**
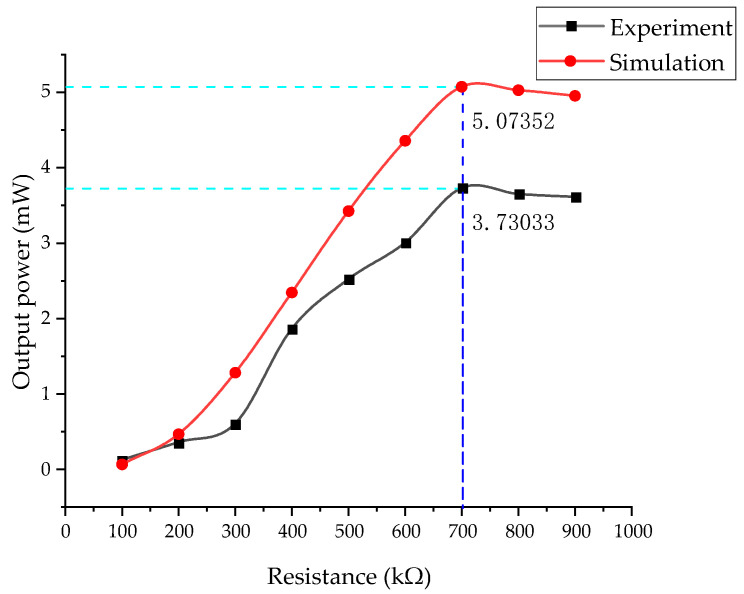
Influence of external resistance on the energy harvester.

The simulation settings of the influence of resistance on voltage and output power are as follows: the boundary load pre-stress, *F*_1_, is 1000 N and the perturbation load, *F*_0_, is 4000 N; step 2: Frequency Domain Perturbation Frequencies: 3 Hz; Auxiliary sweep type: Specified combinations; Parameter name: Resistance; and Parameter value list: range (100, 100, 1000). The optimal resistance of the energy harvester is shown in Figure 11. At the frequency of 3 Hz, the pre-stress is 1 kN and the load is 4 kN. The measured voltage increases with the increase in resistance. The optimal resistance is about 700 kΩ by experiment. Its output power is 3.73 mW. The simulation results show that the optimal resistance is about 700 kΩ and its output power is 5.07 mW. The simulation is in good agreement with the experimental trends.

**Figure 12:**
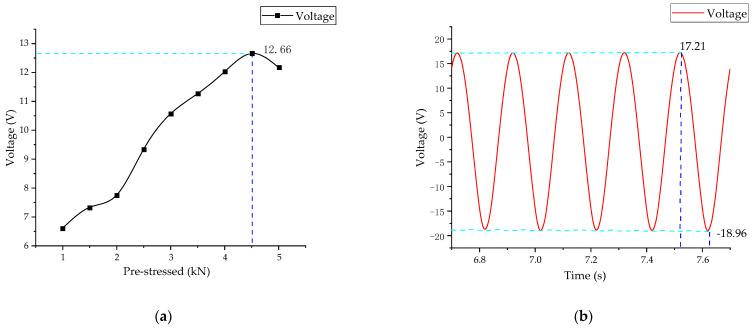
Influence of pre-stress on the output voltage. (**a**) Influence of external pre-stress on output voltage; (**b**) time domain diagram of the output voltage at pre-stress 4.5 kN.

The pre-stress of the energy harvester is shown in Figure 12. Under the excitation of a sinusoidal load of 1 kN at a frequency of 5 Hz, the voltage generated by the energy harvester also increases with the increase in the pre-stress. It reaches a maximum of 12.66 V at around 4.5 kN. Thus, the optimal pre-stress is 4.5 kN. It can be seen that the higher the pre-stress is, the better the performance is. However, there is an optimal pre-stress at a specific frequency and excitation load where the energy harvester can work under these conditions at the highest energy harvesting efficiency. Experiments with load frequency can further verify this in subsequent studies. This provides essential guidance in configuring, placing, and installing energy captors to ensure energy capture efficiency and installation safety.

**Figure 13:**
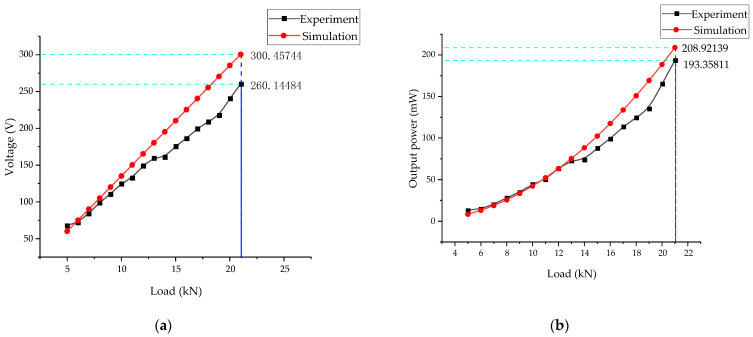
(**a**) Influence of load on the output voltage; (**b**) influence of load on the output power.

The simulation settings of perturbation load on voltage area as follows. The boundary load pre-stress, *F*_1_, is 1000 N, the perturbation load *F*_0_. Study: Frequency Domain, Pre-stress; step 1 is the default; step 2: Frequency Domain Perturbation Frequencies: 3 Hz; Auxiliary sweep type: Specified combinations; Parameter name: *F*_0_; and Parameter value list: range (1000, 1000, 21,000). The load of the energy harvester is shown in Figure 13. Under the frequency of 3 Hz and a pre-stress of 1 kN, the voltage generated by the energy harvester increases significantly with the perturbation load. When the perturbation load is 21 kN, the open-circuit RMS voltage generated in the experiment reaches 260.14 V. The RMS voltage generated by the simulation is 270.41 V. The maximum total output power of the experiment is 193 mW and the simulation result is 208 mW. Based on the track displacement and force analysis, the impact size developed when the wheel passes through the energy harvester is inferred. Then, it is coupled to the load and frequency generated by the energy harvester to obtain the size of energy captured.

Energy efficiency is an essential parameter in evaluating energy harvesters. The input mechanical energy mainly comes from the vibration displacement caused by the qua-si-static load of the train on the track. This paper uses the MTS testing machine to simulate the quasi-static vibration of a railway track, with a set frequency of *f* = 3 Hz and a load of *F* = 21 kN. It can be seen from the above that the stiffness of the energy harvester is *k_e_* = 10 MN/m. Formulas (2)–(5) are shown below to calculate important values.

(2) $$F_{0}=F\sin\left(2\pi ft\right)$$

(3) $$x=\frac{F_{0}}{k_{e}}=\frac{F\sin\left(2\pi ft\right)}{k_{e}}$$

(4) $$V=\frac{dx}{dt}=\frac{2\pi fF}{k_{e}}\cos\left(2\pi ft\right)$$

(5) $$P_{in}=\frac{\int_{0}^{T}F_{0}\cdot Vdt}{T}$$

where *F* is the quasi-static load, *F*_0_ is the sine load, *x* is the displacement, *k_e_* is the stiffness of the energy harvester, *V* is the displacement velocity, *t* is the time, and *T* is the minimum positive period. The mechanical input power is 21 W and the electrical output power is 193 mW, so the energy efficiency is $\eta =\frac{0.193}{21}=0.00919=0.92\%$.

As we can see in Figure 10, Figure 11, Figure 12 and Figure 13, there are some errors in the experiment and simulation. The actual voltage generated by the energy harvester is smaller than the simulation results due to the accumulation of the installation, the assembly of the energy harvester, and the size errors among various parts. The influence of load frequency on voltage shows the same trend between the experiment and simulation. The energy harvester is mainly composed of two arch beams, two support plates, and a base. The machining error is mainly the radius of the arch beam and the flatness of the contact surface with the testing machine. The assembly error is mainly the coaxiality of the two rods. A gap exists between the hole of the rod shaft and the base. The parallelism between the upper and lower planes of the arch beam is also the main component of assembly error. The error of the test machine is mainly reflected in the load force, which is ±100 N.

The above analyzes the influence of different loads on the output of the energy harvester at a fixed frequency. The larger the load in a particular range, the higher the output. Under a fixed load, the influence of different frequencies on the output of the energy harvester is significant when the frequency is 1–6 Hz, but not noticeable when the frequency exceeds 12 Hz. Furthermore, the influence of resistance on output power under a constant vibration environment is determined and the best resistance value range is found. The NI 9229 data acquisition card was used for data acquisition, with an effective range of ±60 V, and the output voltage of the energy harvester exceeded 200 V under large loads. The internal resistance of the acquisition card by measurement is 1 MΩ. To expand the acquisition card’s measuring range, 4 MΩ resistors were connected in series, which increased the measuring range by four times to ±300 V. The optimal resistance of the energy harvester is about 700 kΩ by experiment. When the load is 4 kN and the pre-stress is 1 kN, the output power of the energy harvester is 3.73 mW. The simulation results show that the optimal resistance is about 700 kΩ. The output power is 5.07 mW. The load has the most significant influence on energy capture efficiency. The simulated voltage is 260.14 V and the actual voltage is 270.41 V. The error rate is 3.7%, which correlates with the equivalent stiffness. When the load is 21 kN and the frequency is 3 Hz, the single-side output power is 96.57 mW and the total power is about 190 mW. The power of the ZigBee transmission chip is usually within 40 mW and the acceleration sensor is 55 μW, so it is suitable to use sensors to capture the energy of track vibrations.

## 4. Conclusions

A piezoelectric stack energy harvester was designed and fabricated for railway vibration energy harvesting. Applying the energy harvester arch beam creates a good buffer for the shock vibration caused by the train. The piezoelectric stack is positioned horizontally to avoid direct impact and make full use of the space; thus, it can be adapted to different rail system applications. The performance of the energy harvester is verified by experiments and analyzed by simulations. (1) The proposed design scheme combines an arch beam, a piezoelectric stack, and a rod which are stacked stack horizontally. It places the energy harvester in the railway system with a small vertical space below the track. (2) Through the verification of experimental and simulation analyses, the energy harvester can be adapted to the application scenario of the railway system. It can be used as a basis to guide and optimize the theoretical model. (3) At low frequencies, the output voltage of the energy harvester is affected by an increase in frequency; when the load frequency is higher than 12 Hz, the effect on the output voltage of the energy harvester is small. When the prestress is around 4.5 kN, the energy harvester has a certain assembly stress, so the gap between the joints of each part is controlled to ensure a good energy harvesting effect. (4) In the current simulation environment, the measured weight of the energy harvester is 912 g and the output power is 193 mW. The energy density of the energy harvester is 211.8 μW/g. In a heavy-load environment, the energy harvesting effect increases with the load, and its energy density also increases. This result matched the experimental results from the virtual environment. The energy harvester can be used for train track vibrations, in highway systems, and in other heavy duty, low-frequency environments. Compared to other energy harvesters, it has the advantages of wide applicability, a simple structure, good durability, and a low failure rate. In future work, energy capture devices will be used for networking, to track data collected to monitor trains’ running status through algorithms, and for network analyses of trains and track health status.

## Data Availability

Not applicable.
